# Poly(Acrylic Acid)-Sodium Alginate Superabsorbent Hydrogels Synthesized Using Electron-Beam Irradiation—Part III: An Evaluation of Their Degradation in Soil

**DOI:** 10.3390/molecules30051126

**Published:** 2025-02-28

**Authors:** Elena Manaila, Ion Cosmin Calina, Marius Dumitru, Gabriela Craciun

**Affiliations:** Electron Accelerators Laboratory, National Institute for Laser, Plasma and Radiation Physics, 409 Atomistilor St., 077125 Magurele, Romania; elena.manaila@inflpr.ro (E.M.); calina.cosmin@inflpr.ro (I.C.C.); marius.dumitru@inflpr.ro (M.D.)

**Keywords:** poly(acrylic acid), sodium alginate, electron beam, superabsorbent hydrogels, swelling media, burial in soil, degradation

## Abstract

Global challenges in agriculture, in terms of water and nutrient loss control, require new approaches to maintaining or even increasing crop production. Promising materials, such as superabsorbent hydrogels of hybrid types obtained from natural polymers grafted with synthetic polymers, represent a viable solution to solve these problems and maintain a clean environment. In view of this, two types of hydrogels based on sodium alginate, acrylic acid and polyethylene oxide obtained using 5.5 MeV electron-beam irradiation were subjected to degradation through burial in the soil. Swollen hydrogels in two types of water (distilled and tap) and two types of nutrient solutions (synthetic nutrient solution and 100% natural organic nutrient solution), with different pHs of 5.40, 6.05, 7.45 and 7.66, were buried in soil for 30 and 60 days and then extracted and analyzed in terms of their mass loss, swelling behavior and cross-linking structure. The highest mass losses after both 30 and 60 days were recorded for the hydrogels buried in soils whose humidity was maintained by watering them with the basic solutions (tap water and the organic nutrient solution). Structural modifications associated with the degradation process were highlighted by decreases in the cross-link densities and increases in the mesh sizes and swelling. These results were confirmed using FTIR and SEM techniques.

## 1. Introduction

Climate change; aggressive poaching of agricultural lands; the adoption of unfavorable practices like the use of chemicals; water scarcity; erosion, degradation and nutrient loss in the soil; and population growth represent challenges that impact both the global agricultural landscape and the actions that need to be taken to adapt to these conditions. In addition to these listed above, inadequate rainfall and the ineffective utilization of water represent reasons why the quantity and quality of produced food are affected [1]. In these circumstances, it has become necessary to change the approach to problems related to water scarcity and soil degradation [1,2] by finding and quickly moving towards innovative solutions because productivity in the agricultural field depends, among other things, on soil quality and water availability [1,3,4]. The class of superabsorbent polymer materials/hydrogels has experienced great development due to its potential to solve some of the above problems. Hydrogels are 3D cross-linked polymer networks with special hydrophilic properties that are capable of absorbing large amounts of aqueous solutions (with or without nutrients) and are suitable for improving the soil’s properties [5,6,7,8]. This type of three-dimensional network results from the creation of temporary non-covalent physical bonds, which are reversible and short-lived with poor mechanical properties (through electrostatic interactions, hydrogen bonds, hydrophobic interactions and molecular entanglements) [1,9,10]; permanent covalent chemical bonds (using reactive cross-linking agents such as glutaraldehyde [1,11] or epichlorohydrin [1,12] or polyanions such as sodium triphosphate, sodium oxalate and sodium citrate that form ionic bonds [1,13]); or a combination of these in their matrix structure [8]. To address the needs of the agricultural field (water and nutritional stress in the soil, for example), hydrogels must, in addition to having a high absorption capacity, be permanent and have high mechanical and thermal stability and controllable properties, and these conditions are met only by hydrogels obtained using chemical bonds [10].

Due to their nutrient and water retention capacities, hydrogels can act as transport and slow-release agents for fertilizers in the soil conditioning process [14,15,16,17]. In this way, crop productivity and yield are maintained or increased by improving the soil’s density, structure and permeability due to the infiltration and evaporation processes of the water released by hydrogels applied in the soil [14,16,18,19]. A hydrogel placed in artificially or naturally irrigated (by rain) soil absorbs and retains water, preventing its rapid loss through drainage or evaporation. The soil remains moist for long periods of time because the stored water is released from the hydrogel in a controlled manner through specific diffusion mechanisms. At the same time, the increased size of the hydrogel granules due to water absorption increases the porosity of the soil, which ensures better oxygen circulation to plant roots [16,18].

Initially, most hydrogels were prepared from synthetic polymers using chemical cross-linking techniques, which initially limited their use in applications that required properties such as biodegradability or non-toxicity [20,21,22]. Thus, the most popular hydrogels, used even in agricultural applications, are obtained based on cross-linked polyacrylamide (PAM), polyacrylic acid (PAA) and their salts. These have very good physical and chemical properties and macromolecular dimensions much too large to be absorbed into plant tissues and therefore do not present a danger of bioaccumulation but are insensitive to aerobic and anaerobic microbiological degradation, degrading by only 10–15% per year into water, carbon dioxide and nitrogen compounds [23,24].

In recent years, the class of superabsorbent polymers has expanded due to the development of hydrogels based on biopolymers such as polysaccharides (alginate, carboxymethylcellulose, chitosan, pectin, etc.) and various proteins that are cheap, abundant and biodegradable [20,25,26]. These are strongly hydrophilic but present major disadvantages in terms of their mechanical properties [10,27]. As a necessity, hybrid or semi-synthetic superabsorbent polymeric materials have been developed more and more based on synthetic and natural polymers, which present the superior physico-chemical properties of synthetic materials alongside the biodegradability of biopolymers [28].

The biodegradation of polymers consists of the breakdown of all of their organic components [29,30]. In the soil, this begins with the colonization of the polymer’s surface by microorganisms, followed by its depolymerization into low-molecular-weight compounds, microbial absorption and finally the reduction of organic material into carbon, CO_2_, water and new microbial biomass [29,30,31]. Specifically, biodegradation involves the completion of four main stages: biodeterioration (which occurs when microbial communities, other decomposition factors and/or abiotic factors fragment the polymeric material into smaller pieces), biofragmentation (which involves the cleavage of polymeric molecules, reducing their molecular weight and generating oligomers, dimers and monomers), assimilation (here energy, new biomass and other primary elements and secondary metabolites are produced in the cytoplasm of microorganisms) and mineralization (which involves the oxidation of simple and complex metabolites into CO_2_, N_2_, CH_4_ and H_2_O) [29,32].

Alginate is a polysaccharide abundantly found as a structural component in brown marine algae (Phaeophyceae) or in capsular form in various soil bacteria [33,34]. Hybrid hydrogels based on acrylamide, itaconic acid and acrylic acid grafted onto sodium alginate chains exhibited degradation rates of 63.5% after being buried in soil for 40 days [33,35]. Hydrogels obtained by cross-linking lignosulfonate with sodium alginate and konjac flour showed mass losses associated with biodegradation in soil of 6% after 60 days and 14% after 120 days [33,36]. In many experiments, it has been observed that not only the burial duration of the hydrogel but also the type of soil influences the process and degree of biodegradation [33,35,36].

This work aimed to evaluate the degradation, as part of the biodegradation process, of hybrid hydrogels based on acrylic acid, sodium alginate and polyethylene oxide, with and without the reaction initiator potassium persulfate. These hydrogels were obtained through irradiation with a 5.5 MeV electron beam in the range of 10 to 20 kGy and were previously characterized from a physical, chemical, structural and morphological perspectives in our earlier studies [37,38]. For this purpose, hydrogels swollen in two types of water and two types of nutrient solutions were buried in soil. After 30 and 60 days, they were extracted and analyzed. To evaluate degradation, mass loss, changes in the swelling degree, cross-link density and mesh sizes were assessed, along with structural analysis by FT-IR and morphological analysis by SEM. The data were statistically analyzed using one-way ANOVA with post hoc Fisher LSD Calculator in OriginProV2023.

## 2. Results and Discussion

### 2.1. Hydrogel Degradation Assessment in Soil

#### 2.1.1. Hydrogel Swelling Assessment in Four Swelling Media

Degradation tests were performed by burying hydrogels in soil in their swollen state, as obtained in previous studies [37,38]. These hydrogels are of a hybrid type, based on acrylic acid, sodium alginate and polyethylene oxide, and were synthesized through electron beam irradiation at doses ranging from 10 to 20 kGy. They were prepared without (referred to here as Type I) and with (referred to here as Type II) a reaction initiator—potassium persulfate (PP) [37,38].

The liquid media used for hydrogel swelling included two types of water and two types of nutrient solutions with different pH levels as follows: distilled water (pH = 6.05), tap water (pH = 7.66), a synthetic fertilizer hereinafter referred to as Sol. A (pH = 5.40, 7.5 mL in 1000 mL of distilled water), and an organic fertilizer, hereinafter referred to as Sol. B (pH = 7.45, 60 mL in 1000 mL of distilled water). Hydrogels in disk form (radius = 9.08 ± 0.15 mm) were weighed and immersed in the above swelling media for 72 h. The results regarding their swelling degrees are presented in Table 1. All experiments were conducted in triplicate.

As shown in Table 1, the results are influenced by both the irradiation dose and the presence or absence of the initiator PP. Previous studies on the properties of this type of hydrogels obtained by electron beam irradiation [37,38] have shown that the degree of swelling decreases with an increase in the irradiation dose due to a higher cross-linking degree and a reduction in mesh size. Moreover, the swelling degree is not only affected by irradiation and the presence of the initiator, which promotes the formation of free radicals and active sites responsible for grafting acrylic acid onto the sodium alginate chain, but also by differences in the swelling media, particularly in terms of pH [37,38]. The lowest swelling degrees were observed in Sol. A (acidic pH) and distilled water (pH between acid and neutral) for samples without PP, which had a low degree of cross-linking (0.840 × 10^−3^ mol/cm^3^) at 10 kGy, compared to those obtained at the same dose but with PP, which exhibited a higher degree of cross-linking (1.117 × 10^−3^ mol/cm^3^). Immersion of samples obtained at 15 and 20 kGy in slightly basic environments (Sol. B and tap water) resulted in higher swelling degrees compared to distilled water, particularly for Type II hydrogels. The pH of distilled water is typically 7 immediately after distillation but decreases from 7 to 5.8 upon exposure to air, either due to the absorption of atmospheric gases, including CO_2_, or the presence of trace elements remaining after distillation. Both of these factors can alter the hydrophilicity and electrostatic repulsion of the hydrogel, leading to programmable swelling behavior [39,40,41]. Additionally, differences exist in the affinities of microelements fluorine, iodine, zinc, etc.) and macroelements (calcium, magnesium, potassium salts, chlorides, nitrites, nitrates, etc.) present in tap water and the two nutrient solutions for the hydrogel components, particularly for sodium alginate [39]. The swelling of hydrogels is primarily attributed to the presence of strongly hydrophilic functional groups, specifically carboxylic (-COOH) and hydroxyl (-OH) groups [1,3]. The degree of protonation of carboxylic groups is closely related to the pH of the swelling medium. At higher pH levels, carboxylic groups become negatively charged and more extended, facilitating the diffusion of water molecules into the hydrogel network. In contrast, at lower pH levels, hydroxyl groups are protonated, reducing their polarity, which decreases the hydrogel’s affinity for water [39,42,43]. Furthermore, the presence of hydroxyl (-OH) and carboxyl (-COOH) groups plays a crucial role in improving soil characteristics and enhancing water retention [1].

#### 2.1.2. Hydrogel Mass Loss Assessment in Soil

The ability of hydrogels to degrade under aerobic and anaerobic conditions through microbial enzymatic action is one of their remarkable properties. Moreover, the biodegradation of organic substances is primarily influenced by their water solubility and ability to absorb water or aqueous solution [44]. The degradation of the previously obtained and characterization hydrogels [37,38], which were subjected to swelling as described above, was investigated after 30 and 60 days following their placement in soil. We used a universal soil for plants and flowers with a high organic matter content (29–60%), mineral salts (0.35–0.75%), mineral nitrogen (25–50 ppm), water-soluble potassium (52–85 ppm), soluble phosphorus soluble (32–55 ppm) and neutral pH. Hydrogels in their swollen state, prepared in each of the four swelling media, with known masses, dimensions and shapes, were placed in plastic boxes containing a known quantity of soil, ensuring that the samples were completely covered. Soil temperature and humidity were maintained at constant levels throughout the experiment. Half of the samples were extracted after 30 days, while the remaining half were extracted after 60 days.

**Figure 1:**
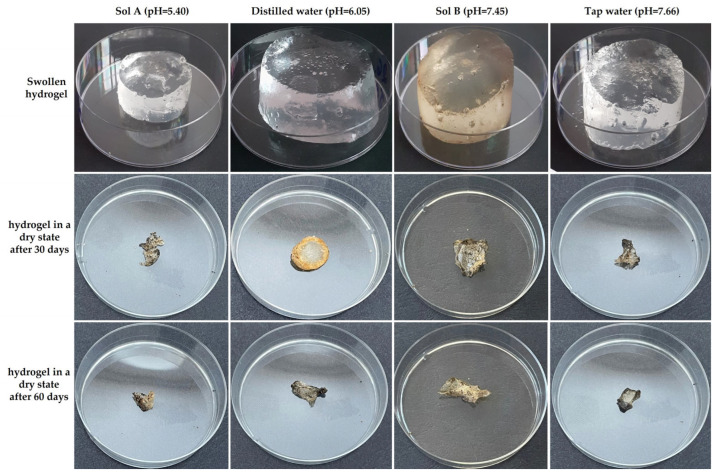
Photographs of hydrogels in their swollen state and dry state after 30 and 60 days.

**Figure 2:**
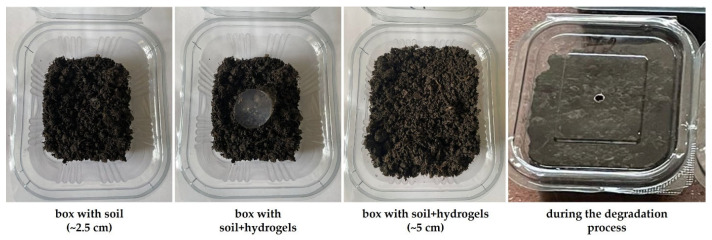
Soil-filled boxes during the degradation experiment.

Photographs of the hydrogels, in their swollen state and dried after extraction from the soil at 30 and 60 days, are shown in Figure 1. Figure 2 presents photographs of the boxes containing soil at the start of the experiment and during its course.

**Figure 3:**
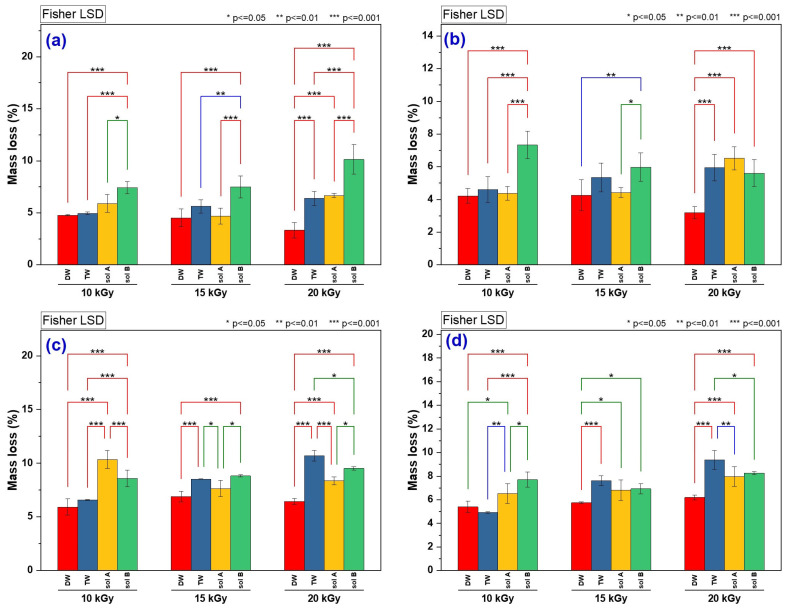
Significant difference between the water types used in the degradation test for irradiation doses applied in hydrogel processing (Fisher’s LSD Multiple Comparison Test) for mass loss after 30 and 60 days: (**a**) mass loss of Type I hydrogel after 30 days, (**b**) mass loss of Type II hydrogel after 30 days, (**c**) mass loss of Type I hydrogel after 60 days, (**d**) mass loss of Type II hydrogel after 60 days.

After 30 and 60 days, the samples were extracted from the soil, washed to remove surface traces of soil, dried at 50 °C for 24 h, and then weighed to determine the mass loss. The results for mass loss are presented in Table 2, while the statistical analysis of the data, using one-way ANOVA and Fisher’s LSD Multiple Comparison Test, are shown in Table 3 and Figure 3.

As shown in Table 2, the mass loss was influenced by the time elapsed between introduction and extraction from the soil. The highest mass losses, after both 30 and 60 days, were observed in the case of hydrogels buried in soils where humidity was maintained by watering with basic solutions (TW and Sol. B).

The statistical analysis was performed with the following purposes: (No. 1) to highlight possible significant differences in terms of mass loss of the hydrogels due to the conditions of their preparation (the three irradiation doses) and the swelling media/wetting of the soil throughout the experiment (DW, TW, Sol. A and Sol. B in Table 3); (No. 2) to identify possible significant differences in the mass loss of hydrogels obtained at the same irradiation dose and swelling/wetting conditions of the soil throughout the experiment. When the *F* value is greater than the *F* critical value, it indicates a significant difference between the group means, suggesting a notable variation between the groups. If *F* value < *F* critical value, the group means are similar, and there may be no significant difference between them. The same reasoning applies to the *p*-value: if *p* < 0.05 (the significance level), there are statistically significant differences between the groups. In other words, there is evidence to reject the null hypothesis, which assumes no significant differences. Conversely, if *p* > 0.05, there is insufficient evidence to reject the null hypothesis, meaning that any observed differences are likely due to the change. From Table 3, it can be observed that for purpose No. 1, the group means are similar, and there may not be significant differences among them: DW (Type I at 30 and 60 days, Type II at 30 days), TW (Type II at 30 days), Sol. A (Type II at 60 days), Sol. B (Type II at 30 days and Type I at 60 days). For purpose No. 2, the group averages are similar and without significant differences only for the Type II hydrogels obtained at the 15 kGy dose and buried for 30 days. For all other samples, significant differences were observed.

One-way factorial analysis of variance (ANOVA) using Fisher’s Least Significant Difference (LSD) Multiple Comparison Test (post hoc test) for the mass loss of hydrogels obtained at the same irradiation dose is presented in Figure 3. Significant pairwise differences in degradation can be observed at both 30 and 60 days for hydrogels obtained at the same irradiation dose and subjected to different swelling/wetting media types.

From Figure 3a, it can be seen that the Type I hydrogels obtained at an irradiation dose of 10 kGy showed significant differences in mass loss upon contact with soil and all four swelling/wetting media at *p* = 0.05 (LSD = 0.98897), and for DW/Sol. B and TW/Sol. B at *p* = 0.001 (LSD = 2.16205). Those obtained at the irradiation dose of 15 kGy exhibited significant differences in mass loss upon contact with TW/Sol. B at *p* = 0.01 (LSD = 2.30993), and with DW/Sol. B and Sol. A/Sol. B at *p* = 0.001 (LSD = 3.47055). The same type of hydrogels obtained at an irradiation dose of 20 kGy showed significant differences in mass loss upon contact with DW/TW, DW/Sol. A, DW/Sol. B, TW/Sol. B and Sol. A/Sol. B at *p* = 0.001 (LSD = 3.60553).

In Figure 3b, Type II hydrogels obtained at an irradiation dose of 10 kGy showed significant differences in mass loss upon contact with soil, and DW/Sol. B, TW/Sol. B and Sol. A/Sol. B at *p* = 0.001 (LSD = 2.68653). Those obtained at the irradiation dose of 15 kGy showed significant differences in mass loss upon contact with Sol. A/Sol. B at *p* = 0.05 (LSD = 1.49127), and with DW/Sol. B at *p* = 0.001 (LSD = 2.16989). The same type of hydrogels obtained at an irradiation dose of 20 kGy showed significant differences in mass loss upon contact with DW/TW, DW/Sol. A, DW/Sol. B at *p* = 0.001 (LSD = 2.89305).

From Figure 3c, it can be seen that the Type I hydrogels obtained at an irradiation dose of 10 kGy showed significant differences in mass loss upon contact with soil and DW/Sol. A, DW/Sol. B, TW/Sol. B and G. A/Sol. B at *p* = 0.001 (LSD = 2.83857). Those obtained at the irradiation dose of 15 kGy showed significant differences in mass loss upon contact with TW/Sol. A and Sol. A/Sol. B at *p* = 0.05 (LSD = 0.85662), with DW/TW and DW/Sol. B at *p* = 0.001 (LSD = 1.87272). Hydrogels obtained at the irradiation dose of 20 kGy showed significant differences in mass loss upon contact with DW/TW, DW/Sol. A, DW/Sol. B, and TW/Sol. at *p* = 0.001 (LSD = 1.43732).

As shown in Figure 3d, Type II hydrogels obtained at an irradiation dose of 10 kGy showed significant differences in mass loss upon contact with soil and DW/Sol. A and Sol. A/Sol. B at *p* = 0.05 (LSD = 1.08679), TW/Sol. A at *p* = 0.01 (LSD = 1.58136), DW/Sol. B and TW/Sol. B at *p* = 0.001 (LSD = 2.37591). Those obtained at the irradiation dose of 15 kGy showed significant differences in mass loss upon contact with DW/Sol. A and DW/Sol. B at *p* = 0.05 (LSD = 0.99684) and DW/TW at *p* = 0.001 (LSD = 2.17926). Hydrogels obtained at the irradiation dose of 20 kGy showed significant differences in mass loss upon contact with TW/Sol. B at *p* = 0.05 (LSD = 1.11521), TW/Sol. A at *p* = 0.001 (LSD = 2.43803), and DW/TW, DW/Sol. A and DW/Sol. B at *p* = 0.001 (LSD = 2.43803).

The biodegradation of the hydrogel begins with the biopolymer chain, which, in this case, is sodium alginate. This process can occur through enzymatic oxidation, which cleaves C-O or C-C bonds, or through the hydrolysis of functional groups such as OH- or CO- [44,45]. Regardless of the degradation mechanism, cleavages in the polymer chain lead to the formation of small molecule compounds and a decrease in molecular weight, which is reflected in the observed mass loss. Additionally, as the biopolymer degrades, its fragments become a source of nutrients for soil microorganisms [44]. However, the biodegradation process is also highly dependent on the characteristics of the soil, such as pH, organic matter content, and the ratio of organic carbon to total nitrogen content, C/N, among others. For example, testing three different types of soil (forest soil with pH = 6.6, C/N ratio = 12.7; coastal soil with pH = 7.8, C/N ratio = 8.8; and agricultural soil with pH = 8.1, C/N ratio = 6.7) to establish a correlation between their properties and the biodegradation of certain hydrogels showed that the soil with the highest pH and the lowest C/N ratio resulted in the highest degree of biodegradability. Additionally, the relationship between the soil’s pH level and the change in the C/N ratio was highlighted. It was demonstrated that an increase in soil pH leads to a decrease in the C/N ratio [45].

Our results show that the highest degradation rates were obtained for hydrogels immersed in solutions with a high pH. After 30 days in soil, hydrated with the same type of solution used for hydrogel swelling, the highest degrees of degradation, 10.14% for Type I and 7.34% for Type II, were observed for hydrogels immersed in Sol. B (pH = 7.45). After 60 days in soil, the highest mass losses were recorded for hydrogels immersed in tap water (10.70% for Type I and 9.38% for Type II) and Sol. B (10.52% for Type I and 8.26% for Type II). Even though tap water did not introduce organic matter into the soil, its high pH has the potential to alter the C/N ratio in the soil [45], thus explaining the comparable results with those obtained from Sol. B, which is a 100% organic product (derived from vermicompost), regardless of the hydrogel type. Vermicompost is a product of organic waste degradation by worms. It has a large specific surface area and good ion exchange and adsorption capacity, and it contains rich nutrients as well as beneficial microorganisms for soil and plants [46,47,48]. The presence of vermicompost increases the content of humic acids and enhances acid phosphatase activity, thereby improving the enzymatic activity of microorganisms [48]. As a result, Sol. B contributes additional microbial activity to the soil, leading to the high biodegradation observed in the tested hydrogels [47].

Table 4 shows the change in mass loss of hydrogels after 60 days compared to 30 days, highlighting the impact of doubling the burial time and correlating the results with the hydrogel type, production conditions and initial properties.

Significant increases in mass loss were observed after doubling the residence time in the soil for hydrogels of both types swollen in distilled water.

After 60 days, mass loss increased by 93.09% for Type I hydrogels obtained at 20 kGy, and 94.04% for Type II hydrogels at the same irradiation dose. While these results are promising, it is important to note that distilled water is not typically used to hydrate plants in real-world conditions. Hydrogels of the same type and irradiation dose (20 kGy), swollen in tap water, exhibited mass losses of 67.45% (Type I) and 57.65% (Type II). This is particularly relevant as tap water is commonly used to maintain soil moisture during plant growth and development. The use of the acidic solution (pH = 5.40), Sol. A, for swelling the hydrogels and hydrating the soil resulted in mass losses after 60 days of 75.25% and 63.68% of the Type I hydrogels obtained at irradiation doses of 10 and 15 kGy. For Type II hydrogels under the same conditions, mass losses were 49.20% and 54.30%. The results are significant, considering these hydrogels had lower degrees of cross-linking compared to those obtained at 20 kGy. Doubling the residence time in soil for hydrogels swollen in Sol. B resulted in the best degradation rates, particularly for Type II hydrogels obtained at 20 kGy, with a mass loss of 47.24%. This is notable as the stability of these hydrogels suggests their potential for use in multiple swelling/deswelling cycles [49].

#### 2.1.3. Hydrogel Cross-Link Density, Mesh Size and Swelling at Equilibrium Assessment After Burial in Soil

The gradual biodegradation of a material due to specific biological activity is accompanied by the deterioration of its molecular structure [33,50]. This process occurs in three stages: biodeterioration, biofragmentation and bioassimilation [51,52]. In the first stage, superficial degradation occurs, leading to modification in the material’s physical, chemical and mechanical properties. This process generally takes place under abiotic conditions when environmental factors such as heat, light, temperature, humidity, solvents and mechanical compressions weaken or damage the material. These changes may be visible to the naked eye, such as alterations in color, size, integrity and mass, or they may require specialized instruments for detection, such as variations in mechanical properties, rheological behavior, crystallinity, oxidation state or molecular mass distribution [53]. In parallel, biotic biodeterioration also occurs, involving microbial action (bacteria, fungi) that contributes to the biofragmentation of the material. Biofragmentation is the stage in which high-molecular-weight molecules and polymeric structures, resulting from cross-linking, polymerization or grafting reactions, are degraded either by bond cleavage (due to enzymatic action) or by chemical modification. As a result, oligomeric or monomeric fragments are formed. This oxidation–reduction process requires energy and can take place both in the presence of oxygen (aerobic) and in its absence (anaerobic). Bioassimilation is the last stage of biodegradation, during which microbes or microorganisms present in the soil utilize the bio-fragmented material in their metabolic activities, either for energy generation (adenosine triphosphate, ATP) or as a precursor for other biosynthetic pathways [51].

In the biodegradation process of hydrogels buried in soil, the first step involves colonization by microorganisms that release enzymes necessary for breaking down molecules into smaller compounds, such as oligomeric and monomeric fragments [30,33,54]. Since the tested hydrogels underwent the biodegradation process after swelling, with humidity being maintained constant throughout the burial period using the same type of swelling medium, degradation in this case is also influenced by the presence of water, making it predominantly hydrolytic. In hydrolytic degradation, water molecules in the swelling media (aqueous) react with functional groups in the polymer structure, such as esters, amides or ethers, leading to the cleavage of the polymer chains. As a result, shorter chains are formed, which then detach from the hydrogel network due to diffusion [33,54]. The two types of degradation (microbial and hydrolytic) contribute to the deterioration process through cleavage and depolymerization reactions, ultimately leading to changes in the material’s properties. The gradual breaking of polymer chains and the cross-linked structure results in the formation of rough surfaces, cracks and holes in the material, which are characteristic signs of degradation [33,55]. As a consequence of these cleavage reactions, the network parameters of the hydrogels undergo irreversible changes.

The values of certain structural properties after 30 and 60 days of burial in soil, specifically, cross-linking degree (measured by cross-link density), mesh size and swelling degree (swelling at equilibrium) are presented in Table 5, Table 6 and Table 7.

As seen in Table 5, Table 6 and Table 7, the hydrogels underwent structural modification, evidenced by decreases in cross-link densities and increases in mesh size and swelling after biodegradation. These changes varied depending on the hydrogel type (with or without PP), the conditions under which they were obtained (irradiation dose) and the swelling and watering conditions (distilled water, tap water, Sol. A and Sol. B). After both burial periods, Type I hydrogels exhibited greater decreases in cross-link density compared to Type II hydrogels (Table 5). It appears that, regardless of the radiation dose, the addition of the initiator leads to the formation of stronger bonds that are more difficult to break. However, it is important to note that after 30 days, the modest mass losses of both hydrogel types obtained at an irradiation dose of 20 kGy (3.33% for Type I and 3.19% for Type II, in distilled water) were accompanied by significant decreases in cross-linking density (80.66% and 70.13%, respectively). Conversely, higher mass losses (10.14% for Type I and Type II in Sol. B and 6.52% for Type II in Sol. A) were associated with smaller decreases in cross-linking density (72.63% and 73.75%, respectively). From the perspective of cross-link density reduction, which is associated with the degradation of the polymer structure, the best results for Type I hydrogels were observed as follows: in distilled water, tap water and Sol. B, for hydrogels obtained at 20 kGy (80.65%, 84.35% and 72.62%, respectively) and in Sol. A, for hydrogels obtained at 15 kGy (76.53%). The Type II hydrogels obtained at a dose of 20 kGy exhibited the highest reductions in cross-link density across all four swelling media. Notably, the smallest decreases in cross-link density were observed in two Type II hydrogels obtained at 10 kGy, which were buried for 30 days and hydrated with distilled water (8.06%) and Sol. B (9.40%), respectively. Even after 60 days in soil, the hydrogels obtained at an irradiation dose of 20 kGy, which exhibited the lowest mass losses (6.43% for Type I and 6.9% for Type II, in distilled water), still showed significant reductions in cross-linking density (86.88% and 70.34%, respectively), comparable to those obtained after 30 days of soil incubation. Thus, after 60 days, the most substantial decreases in cross-linking density were observed in Type I hydrogels obtained at 20 kGy, in distilled water, tap water and Sol. B (86.68%, 86.60% and 77.67%, respectively). In Sol. A, the highest reduction was recorded for hydrogels obtained at 15 kGy (77.48%). For Type II hydrogels, the greatest reductions in cross-linking density were observed in those obtained at an irradiation dose of 20 kGy across all four types of swelling media.

Mesh size (ξ, nm) represents the average distance between two neighboring network junctions connected by a polymer chain in a hydrogel and is thus dependent on the cross-linking degree. A decrease in cross-linking degree leads to an increase in mesh size, whereas an increase in the cross-linking degree results in a decrease in mesh size. As shown in Table 6, the greatest changes in mesh size were observed in the hydrogels with the most significant reduction in cross-linking degree. This includes hydrogels obtained at 20 kGy in distilled water, tap water and Sol. B, as well as those obtained at 15 kGy in Sol. A, regardless of the period spent in the soil.

Hydrogel swelling is dependent on the molecular structure, specifically the degree of cross-linking, which in turn affects the mesh size. As seen in Table 7, the largest changes in swelling degree compared to the control (native) samples were observed for the hydrogels obtained at 20 kGy, both after 30 days and 60 days in soil.

Increasing the burial time from 30 to 60 days does not necessarily lead to a doubling of the values for the properties correlated with the bio(deterioration) of the hydrogels, as shown in Table 8.

From Table 8, it can be observed that the best values for the parameters associated with degradation were obtained as follows: Type I hydrogels obtained at 20 kGy in distilled water, Sol. B and tap water, and at 10 kGy in Sol. A; Type II hydrogels obtained at 10 kGy in distilled water, at 15 kGy in Sol. A, and at 20 kGy in Sol. B and tap water.

### 2.2. FTIR

**Figure 4:**
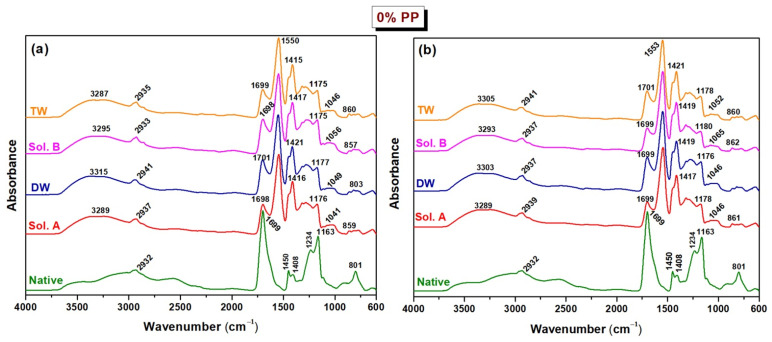
FTIR-ATR spectra of degradation for Type I hydrogels (0% PP) at (**a**) 30 days and (**b**) 60 days. In legends: Sol. A, DW, Sol. B and TW represent pH = 5.4, pH = 6.05, pH = 7.45 and pH = 7.66.

**Figure 5:**
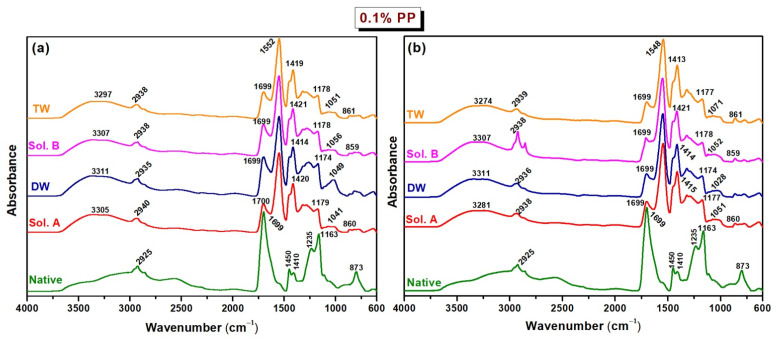
FTIR-ATR spectra of degradation for Type II hydrogels (0.1% PP) at (**a**) 30 days and (**b**) 60 days. In legends: Sol. A, DW, Sol. B and TW represent pH = 5.4, pH = 6.05, pH = 7.45 and pH = 7.66.

The analyses were performed on hydrogels of both types cross-linked at the irradiation dose of 20 kGy (referred to as native) and on samples subjected to long-term incubation in soil for 30 and 60 days, in order to assess their stability and degradation behavior. Figure 4 and Figure 5 illustrate the FTIR spectra obtained for these samples.

According to the FTIR spectra of native hydrogels without an initiator, Type I, the strong absorption bands at 1688–1692 cm^−1^ specific to acrylic acid confirm the presence of the –C=O groups. The bands in the 1409–1450 cm^−1^ range represent the bending vibration for the CH_2_ and CH_3_ groups, while the band at 1233 cm^−1^ is due to the O-H bending vibration. All these bands indicate the cross-linking between acrylic acid, sodium alginate and polyethylene oxide in the hydrogel. For the native hydrogel with 0.1% PP, Type II, two new peaks are confirmed, at 3191 cm^−1^ and 1547 cm^−1^ corresponding to O–H stretching vibrations to sodium alginate and C=O stretching vibrations specific to acrylic acid and sodium alginate. Moreover, with the addition of PP, after the cross-linking of the hydrogels, the presence of the peak at 1057 cm^−1^ is attributed to sulfonic groups, indicating the strong interactions between the polymer components [37].

The spectra in Figure 4 for the Type I hydrogel before degradation (native) show the main bands at 2932 cm^−1^ (C–H), 1699 cm^−1^ (–C=O), 1408–1450 cm^−1^ (CH_2_ and CH_3_), 1234/1184 cm^−1^ (C–O, O–H of carboxylic groups), 1063 cm^−1^ (C–O–C) and 800–850 (CH_2_ rocking mode). The degradation of Type I hydrogel samples swollen in different solutions (Figure 4a,b) is accompanied by a decrease in the intensity of the bands observed in the regions 2900–2950 cm^−1^ and 1200–1450 cm^−1^. Furthermore, a significant reduction in the intensity of the absorption band corresponding to C–H stretching vibrations at 1699 cm^−1^ was noted, with this effect becoming increasingly prominent after 30 and 60 days of burial in soil. This observation suggests potential oxidative degradation of the hydrogel structure, a result that is consistent with those obtained in experiments assessing mass loss and cross-link density after burial in soil. The peak at 2932 cm^−1^ exhibits a redshift, reaching 2941 cm^−1^ under acidic conditions in distilled water (DW) after 30 days and under basic conditions in tap water (TW) after 60 days. Additionally, new absorption bands emerge in the regions of 1550–1555 cm^−1^ and 1275–1285 cm^−1^ across all pH conditions. Notably, the peak at 1117 cm^−1^ remains unchanged and persists in TW after 30 days. Additionally, the intensities of the bands at 1163 cm^−1^, associated with the stretching vibrations of C–O–C groups, decreased markedly across all compositions as degradation progressed, indicating structural damage to the polymer network and potential fragmentation of the macromolecular chains. In the spectra of hydrogels degraded in soil wetted with DW (pH 6.05) for 30 days and 60 days, the CH_2_ rocking bands at 800–850 cm^−1^ disappeared.

The FTIR spectrum of the Type II native hydrogel, as illustrated in Figure 5, exhibits several characteristic absorption bands, providing insights into its molecular structure. A peak at 2925 cm^−1^ corresponding to C–H stretching and a distinct peak at 1699 cm^−1^ are attributed to C=O stretching vibrations, usually associated with carbonyl functional groups. In addition, the presence of CH_2_-related vibrations is evident at 1450 cm^−1^, while CH_2_ deformation vibrations appear at 1410 cm^−1^ and 1339 cm^−1^. The absorption bands at 1235 cm^−1^ and 1163 cm^−1^ are attributed to C–O stretching and O–H vibrations of the carboxylic functional groups, and the peak at 873 cm^−1^ corresponds to the CH_2_ rocking mode, further characterizing the structural composition of the hydrogel.

During the degradation period, Type II hydrogels (Figure 5a,b) showed significant reductions in the absorption bands intensities, observed in the spectral regions corresponding to the C–H, C–O, and O–H functional groups, specifically within the ranges of 2500–2950 cm^−1^ and 1300–1100 cm^−1^. These changes indicate a decrease in the presence of these groups, likely due to chemical degradation, suggesting structural modifications of the polymer chain. Additionally, the intensity and position of the band at 1339 cm^−1^, attributed to CH_2_ bending vibrations, explicitly decreased to 1317 cm^−1^ under basic pH conditions in tap water (TW) as degradation progressed. The absorption band at 1450 cm^−1^ is exclusively observed in the case of DW use. For the Type II hydrogels, the disappearance of bonds at 1235 cm^−1^ and 1115 cm^−1^, corresponding to C–O bond vibrations, suggests significant degradation of this composition. This pronounced loss indicates a high susceptibility to structural breakdown. The increase in the carboxyl group frequency from 1163 cm^−1^ to 1180 cm^−1^ was more pronounced on day 60 in the case of Sol. B use. Moreover, the reduction of the band at 873 cm^−1^, attributed to CH_2_ rocking plane vibrations, and its complete disappearance in DW (pH = 6.05) after 60 days, may be associated with the progressive shortening of polymer chains. This observation suggests that the acidic environment accelerates the hydrogel’s degradation, leading to more extensive polymer fragmentation over time. The spectral positions of the ν(C–H) vibrational bonds and the corresponding Δν values, representing the average frequency shifts before (native) and after degradation, are systematically presented in Table 9. The Type I hydrogels, when incubated in alkaline environments (Sol. B and TW), exhibited a more pronounced frequency shift after 60 days compared to those maintained in acidic conditions. In contrast, for the Type II hydrogels, the band shifts induced by sample degradation over time were less pronounced. However, the cross-linked hydrogels (Type II) demonstrated more significant variations in average vibrational frequencies across different pH conditions, suggesting altered degradation kinetics.

The hydrolysis process of the hydrogel is significantly accelerated by the increased absorption of water within its structure. As water molecules penetrate the hydrogel matrix, they facilitate the cleavage of covalent bonds, weakening the polymer network. This enhanced bond-breaking process leads to a more rapid degradation of the hydrogel, ultimately impacting its stability and structural integrity over time [56].

### 2.3. SEM

**Figure 6:**
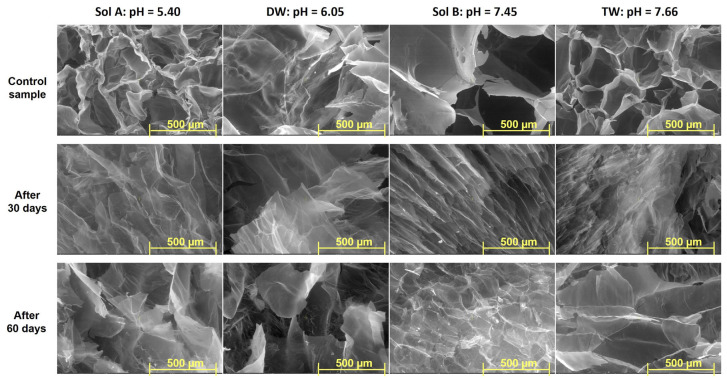
SEM images of Type I hydrogels obtained at 20 kGy: before (control sample), after 30 days and after 60 days in soil (magnification 100×).

**Figure 7:**
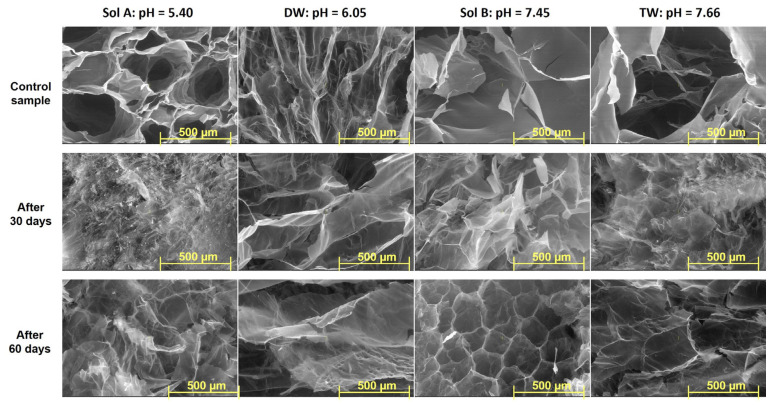
SEM images of Type II hydrogels obtained at 20 kGy: before (control sample), after 30 days and after 60 days in soil (magnification 100×).

**Figure 8:**
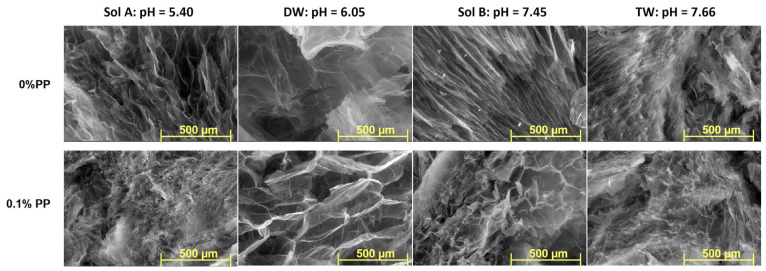
SEM images of Type I and II hydrogels obtained at 20 kGy, after 30 days in soil (magnification 50×).

**Figure 9:**
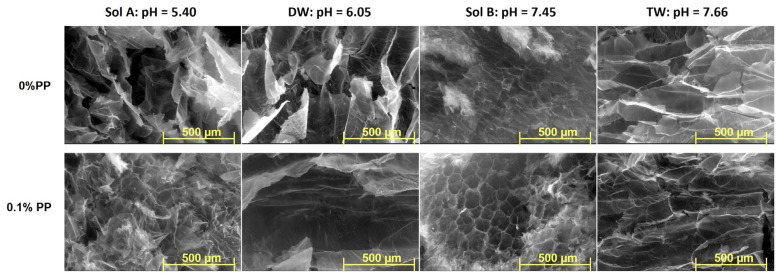
SEM images of Type I and II hydrogels obtained at 20 kGy, after 60 days in soil (magnification 50×).

The Scanning Electron Microscopy (SEM) technique has been used to investigate the inner morphology of hydrogels in their freeze-drying state. Images detailing both types of hydrogels obtained at 20 kGy, before and after burial in soil, with magnification of 50× and 100×, are presented in Figure 6, Figure 7, Figure 8 and Figure 9.

Differences in the inner structures of swollen hydrogels due to the addition of PP in any of the swelling media can be observed in terms of pore sizes and shapes (Figure 6 and Figure 7). The shape and size of the pores, as well as the thickness of the walls after swelling in solutions with increasing pH, are different. This is assumed to influence both the diffusion of the solutions within the polymer network and the way they are subsequently released into the soil where the hydrogels were buried to evaluate degradation (Figure 6 and Figure 7) [37,38]. However, a maintenance of the regularity of the pores can be observed when solutions with low pH were used, in which case their walls also appear firmer.

As is plainly visible in the photographs (Figure 6, Figure 7, Figure 8 and Figure 9), the size and shape of the hydrogel pores change through exposure to the soil environment over time, undergoing visible modification during the process, even after 30 days [57].

Except for the experiments in which distilled water was used, modifications associated with degradation, such as fragmentation, can be easily distinguished, especially in Type II hydrogels used in the experiments using Sol. A and tap water, even after just 30 days (Figure 6, Figure 7, Figure 8 and Figure 9) [57]. The loss of physical integrity may be due to the presence of polysaccharides in the hydrogel, which increases the surface area available for colonization by microorganisms, thereby hastening biodegradation [29,30,31,57]. On the other hand, the pH of the swelling media involved in the experiments may either favor or obstruct degradation [57].

## 3. Materials and Methods

The materials used in the experiments are hydrogels obtained by electron-beam irradiation at 10, 15 and 20 kGy, based on an already presented recipe [37,38,49] and characterized in terms of physico-chemical, structural and morphological properties as described in our previous works [37,38,49]. Ionizing radiation exposure was carried out using a linear accelerator with 5.5 MeV, ALID 7, located in the Electron Accelerators Laboratory at the National Institute for Lasers, Plasma and Radiation Physics, Bucharest, Romania. Radiation dosimetry was performed using a graphite calorimeter, provided by DTU Health Tech, High Dose Reference Laboratory, Roskilde, Denmark, which serves as the primary standard for electron beams [37,38,49]. For hydrogel preparation, chemicals from Merck KGaA, Darmstadt, Germany were used without any further modification: acrylic acid at a concentration of 20% (M_w_ = 71.08 g/mol, density = 1.13 g/cm^3^), sodium alginate at 0.5% (M_w_ = 120,000–190,000 g/mol, viscosity = 15–25 cP, 1% in water), potassium persulfate (PP) at 0.1% (M_w_ = 270.322 g/mol, density = 2.477 g/cm^3^) as a reaction initiator, and polyethylene oxide at 0.1% (M_w_ = 300,000 g/mol, density = 1.210 g/cm^3^) to improve the physical and mechanical properties of the hydrogels [37,38,49].

Hydrogels without (Type I) and with (Type II) PP, characterized as in our previous works [37,38,49], were used for the experiments. Specific properties that were of interest for the present work are presented in Table 10.

In addition, we mention that the gel fraction (%) and porosity (%) ranges were 93.39 ± 2.37–94.48 ± 1.94 and 98.31 ± 0.09–99.51 ± 0.02, respectively, for the Type I hydrogels and 91.79 ± 3.30–94.48 ± 1.94 and 98.61 ± 0.10–99.57 ± 0.0, respectively, for the Type II hydrogels.

### 3.1. Method of Hydrogel Swelling Determination

Four swelling media with different pH values, two types of water (distilled and tap) and two nutrient solutions (Sol. A and Sol. B) were used in swelling experiments before and after the burial in soil. Distilled water was obtained using a laboratory double distiller, tap water was of the potable type from the Magurele, Romania, town network, and nutrient solutions, Sol. A (synthetic) and Sol. B (organic), were purchased from specialty stores in Romania. Some relevant specifications of distilled water, tap water and nutrient solutions are presented in Table 11 [37,38,49].

The preparation of hydrogels for burial in soil, aimed at evaluating their degradation after 30 and 60 days, involved bringing them into a swollen state by immersing them in each of the four swelling media mentioned above. For this purpose, hydrogels with known mass, cut into a disk shape with a diameter of 9.08 ± 0.15 mm, were subjected to swelling for 72 h. The degree of swelling was determined before burial in soil (see Figure 1). The swelling degree was calculated using the following Equation (1) [58,59,60].

(1) $$S\left(\%\right)=\frac{W_{t}-W_{i}}{W_{1}}\times 100,$$

where *W_t_* and *W_i_* are the masses of swollen hydrogels at time *t* and initial in a dry state [37,49].

All experiments were performed in triplicate.

### 3.2. Method of Mass Loss Determination

The hydrogels prepared as described above were buried in plastic boxes containing equal amounts of the same soil. The samples were placed halfway up the soil column, with the height of the column being 5 cm (see Figure 2). The soil used for the experiments was purchased from the Romanian market, and its relevant characteristics are presented in Table 12. The degradation process was monitored after 30 and 60 days, with mass loss being the first determination performed on hydrogels extracted from the soil, using the method that has been adopted for decades [44,61].

Throughout the experiment, the soil humidity was maintained constant by periodic watering using the same swelling media in which the hydrogels had initially been swollen.

For the determination of mass loss as a part of biodegradability process, samples extracted after 30 and 60 days were washed to remove mud from the surfaces and then dried in a laboratory oven at 55 °C until a constant mass was reached and subsequently weighed. The mass loss was calculated using the following equation [44,61,62]:

(2) $$Mass\;loss\left(\%\right)=\frac{W_{i}-W_{d}}{W_{i}}\times 100,$$

where *W_i_* and *W_d_* are the masses of hydrogels before and after burial in soil, in a dried state.

All experiments were performed in triplicate.

### 3.3. Methods of Cross-Link Density, Mesh Size and Swelling at Equilibrium Determination

Equations (3)–(7) were used to evaluate the cross-linked network structure of hydrogels in terms of cross-link density (*q*) and mesh size (*ξ*). To achieve this, the ratio between the molecular weight of the polymer repeating units (*M_r_*), the average molecular weight between cross-links (*M_c_*), and the Flory–Huggins interaction parameter (*χ*) were calculated [37,59,63,64,65].

(3) $$q=\frac{M_{c}}{M_{r}}\;,$$

(4) $$M_{r}=\frac{\left(m_{NaAlg}\times M_{NaAlg}\right)+\left(m_{AA}\times M_{AA}\right)+\left(m_{PEO}\times M_{PEO}\right)}{m_{NaAlg}+m_{AA}+m_{PEO}},$$

(5) $$M_{C}=-V_{1}d_{P}\frac{\nu_{S}^{\frac{1}{3}}-\frac{\nu_{S}}{2}}{\ln(1-\nu_{S})+\nu_{S}+\chi \nu_{S}^{2}},$$

(6) $$\chi =0.431-0.311\nu_{S}-0.036\nu_{S}^{2},$$

(7) $$\xi =\upsilon_{s}^{-1/3}\cdot l\sqrt{\frac{2C_{n}M_{c}}{M_{r}}},$$

where *V*_1_ is the molar volume of the swelling agent (distilled water, 18 cm^3^/mol); *d_p_* is the density of hydrogel; *υ_s_* (cm^3^) is the polymer volume fraction in the swollen state; *l* is the length of the C–C bond (0.154 nm); and *C_n_* is the Flory characteristic ratio of acrylic acid (AA) = 6.7 [66], sodium alginate (NaAlg) = 21.1 [67] and polyethylene oxide (PEO) = 4.98 [68].

The hydrogel swelling at equilibrium (*S_eq_*) was calculated using the Equation (8) [59,63]:

(8) $$S_{eq}(\%)=\frac{W_{eq}-W_{0}}{W_{0}}\times 100,$$

where *W_eq_* and *W*_0_ are the masses of hydrogels at equilibrium and initial after degradation, in a dried state.

All experiments were performed in triplicate.

The results were statistically analyzed using one-way factorial analysis of variance (ANOVA) with post hoc FisherLSD calculator (in the analysis of variance *p* was chosen to be under 0.05) using Microsoft Excel 2021 and OriginPro V2023.

### 3.4. Structural Characterization by FT-IR Technique

Functional groups, before and after degradation, of sodium alginate and acrylic acid in the hydrogels were investigated by FTIR measurements of freeze-dried samples (32 scans/sample) using the Spectrum 100 instrument with a diamond crystal (Perkin Elmer, Waltham, MA, USA) in Attenuated Total Reflectance (ATR) mode at a resolution of 4 cm^−1^ and in the range of 4000–600 cm^−1^. All spectra were analyzed using Spectrum v. 6.3.2 software [37,38].

### 3.5. Morphological Characterization by SEM Technique

Lyophilized hydrogels, both before and after burial in soil, were examined using the Scanning Electron Microscopy (SEM) technique with an FEI/Phillips scanning electron microscope (Hillsboro, OR, USA) [37,38].

## 4. Conclusions

The degradation of two types of hydrogels, obtained through electron beam irradiation at 5.5 MeV, was evaluated in terms of mass loss and structural modification. The experiments involved burying the hydrogels in their swollen state in soil for 30 and 60 days. The swelling media used were selected to represent a range of pH values, from acidic to basic. These included distilled water (pH = 6.05, commonly used for laboratory tests), tap water (pH = 7.66, occasionally used for soil hydration) and two types of nutrient solutions (a synthetic solution with a pH of 5.4, and 100% organic solution with a pH of 7.45). These media are typically employed to address water and nutrient deficiencies in the soil. During the entire burial period, the soil was kept hydrated with the same water or aqueous solution used as the swelling medium before burial. The degradation of the hydrogels was assessed by an evaluation of mass loss, changes in swelling degree, cross-link density, mesh sizes, structure and morphology. The recorded and calculated data were statistically analyzed using one-way ANOVA with post hoc Fisher LSD tests, conducted with OriginProV2023.

The lowest swelling degrees were observed in hydrogels with lower cross-linking (obtained at 10 kGy) and extracted from soils watered with acidic media. In contrast, hydrogels with higher degrees of cross-linking (obtained at 15 and 20 kGy) exhibited significantly higher swelling degrees when extracted from soils watered with basic media. This suggests that an increase in pH facilitates the diffusion of water molecules into the hydrogel network.

A similar trend was observed for mass loss: both after 30 days and after 60 days, the highest mass losses were recorded for hydrogels buried in soils where humidity was maintained using basic solutions.

Soil burial led to structural modification in hydrogels, including decreases in cross-link density and increases in mesh sizes. These two key parameters, which influence the water diffusion mechanism, were affected by multiple factors, including hydrogel type (with or without the reaction initiator—potassium persulfate), synthesis conditions (irradiation dose), and soil watering conditions (distilled water, tap water, synthetic, and organic nutrient solutions). After 30 days, hydrogels of both types obtained at an irradiation dose of 20 kGy exhibited modest mass losses in distilled water (3.33% and 3.19%, yet these were accompanied by significant decreases in cross-linking density (80.66% and 70.13%, respectively). Conversely, when immersed in the organic nutrient solution, the same hydrogels experienced higher mass losses (10.14% for both types) but showed comparatively smaller decreases in cross-linking density (72.63% and 73.75%). From a structural degradation perspective, the most significant reductions in cross-link density were observed in hydrogels obtained without a reaction initiator at 20 kGy, particularly in distilled water (80.65%), followed by tap water (84.35%) and the organic nutrient solution (72.62%). Similarly, hydrogels obtained at 15 kGy showed a notable decrease in cross-link density (76.53%) when exposed to the synthetic nutrient solution. The hydrogels synthesized with a reaction initiator at 20 kGy showed the highest reductions in cross-link density across all four swelling media. In conclusion, the best values of the parameters associated with degradation were obtained as follows: (1) hydrogels obtained without a reaction initiator at 20 kGy, used in distilled water, organic nutrient solution and tap water, and at 10 kGy, used in the synthetic nutrient solution; (2) hydrogels obtained with a reaction initiator at 10 kGy, used in distilled water; at 15 kGy, in synthetic nutrient solution; and at 20 kGy, used in organic nutrient solution and tap water.

FTIR analysis has shown that hydrogels cross-linked with a reaction initiator demonstrated significant variations in average vibrational frequencies under different pH conditions, indicating changes in degradation kinetics.

SEM investigations showed that the size and shape of the hydrogels’ pores changed due to burial in soil, undergoing visible modification even after 30 days. Fragmentation, associated with degradation, was observed in the hydrogels obtained with a reaction initiator when used in the experiments with an organic nutrient solution, even after only 30 days. The pH of the swelling media used in the experiments influenced the degradation process, either facilitating or inhibiting it.

## Figures and Tables

**Table 1:** Swelling degree of hydrogels after 72 h (%).

Swelling Media	0% PP (Type I)			0.1% PP (Type I)		
	10 kGy	15 kGy	20 kGy	10 kGy	15 kGy	20 kGy
	0% PP (Type I)					
Sol. A (pH = 5.40)	4119 ± 231 ^abC^	4655 ± 234 ^aC^	3747 ± 221 ^bC^	5965 ± 401 ^aC^	4975 ± 240 ^bC^	3700 ± 202 ^cC^
Distilled water (pH = 6.05)	13,244 ± 730 ^aA^	9160 ± 266 ^bB^	3981 ± 255 ^cC^	17,066 ± 1138 ^aA^	8562 ± 565 ^bB^	4768 ± 352 ^cB^
Sol. B (pH = 7.45)	10,500 ± 474 ^bB^	11,943 ± 461 ^aA^	7639 ± 411 ^cA^	12,139 ± 473 ^aB^	10,740 ± 311 ^bA^	8573 ± 496 ^cA^
Tap water (pH = 7.66)	10,672 ± 408 ^aB^	8404 ± 419 ^bB^	6040 ± 333 ^cB^	11,341 ± 775 ^aB^	9079 ± 446 ^bB^	7732 ± 459 ^bA^

Values within each row for each data set with different lower letters superscripts are significantly different (*p* ≤ 0.05). Values within each column for each data set with different upper letters superscripts are significantly different (*p* ≤ 0.05).

**Table 2:** Mass loss (%) of hydrogels after 30 and 60 days.

Swelling Media	30 Days			60 Days		
	10 kGy	15 kGy	20 kGy	10 kGy	15 kGy	20 kGy
	0% PP (Type I)					
Sol. A (pH = 5.40)	5.90 ± 0.86 ^abB^	4.68 ± 0.77 ^bB^	6.67 ± 0.20 ^aB^	10.34 ± 0.85 ^aA^	7.66 ± 0.75 ^bAB^	8.36 ± 0.38 ^bC^
Distilled water (pH = 6.05)	4.77 ± 0.05 ^aB^	4.52 ± 0.87 ^aB^	3.33 ± 0.74 ^aC^	5.92 ± 0.75 ^aB^	6.90 ± 0.50 ^aB^	6.43 ± 0.27 ^aD^
Sol. B (pH = 7.45)	7.42 ± 0.58 ^bA^	7.49 ± 1.04 ^abA^	10.14 ± 1.42 ^aA^	8.59 ± 0.79 ^aA^	8.82 ± 0.10 ^aA^	10.52 ± 0.16 ^aB^
Tap water (pH = 7.66)	4.95 ± 0.14 ^bB^	5.62 ± 0.64 ^abAB^	6.39 ± 0.67 ^aB^	6.58 ± 0.05 ^cB^	8.54 ± 0.02 ^bA^	10.70 ± 0.49 ^aA^
	**0.1% PP (Type II)**					
Sol. A (pH = 5.40)	4.37 ± 0.42 ^bB^	4.42 ± 0.31 ^bA^	6.52 ± 0.71 ^aA^	6.52 ± 0.83 ^aAB^	6.82 ± 0.87 ^aAB^	7.97 ± 0.84 ^aA^
Distilled water (pH = 6.05)	4.22 ± 0.45 ^aB^	4.26 ± 0.94 ^aA^	3.19 ± 0.38 ^aB^	5.41 ± 0.47 ^bBC^	5.75 ± 0.07 ^abB^	6.19 ± 0.20 ^aB^
Sol. B (pH = 7.45)	7.34 ± 0.84 ^aA^	5.98 ± 0.87 ^aA^	5.61 ± 0.82 ^aA^	7.70 ± 0.64 ^abA^	6.93 ± 0.44 ^bAB^	8.26 ± 0.12 ^aA^
Tap water (pH = 7.66)	4.60 ± 0.78 ^aB^	5.35 ± 0.88 ^aA^	5.95 ± 0.81 ^aA^	4.94 ± 0.06 ^cC^	7.63 ± 0.41 ^bA^	9.38 ± 0.80 ^aA^

Values within each row for each data set with different lower letters superscripts are significantly different (*p* ≤ 0.05). Values within each column for each data set with different upper letters superscripts are significantly different (*p* ≤ 0.05).

**Table 3:** One-way ANOVA test results: *F*-statistic and *p*-value.

Source	Type I				Type II			
	30 Days		60 Days		30 Days		60 Days	
	*F* Value	*p* Value	*F* Value	*p* Value	*F* Value	*p* Value	*F* Value	*p* Value
Sol. A	6.55041	0.031	12.158	0.00775	17.47761	0.00314	2.43607	0.16808
DW	4.13098	0.07446	2.43342	0.16832	2.68745	0.14676	5.19525	0.04905
Sol. B	6.27388	0.03385	3.17573	0.11463	3.47624	0.0994	6.4635	0.03186
TW	5.27011	0.04773	159.34982	<0.0001	2.01043	0.21465	55.81697	1.32695 × 10^−4^
10 kGy	15.94506	9.75789 × 10^−4^	25.44076	1.91662 × 10^−4^	15.37189	0.0011	13.72213	0.00161
15 kGy	7.82764	0.00916	11.08274	0.0032	3.12963	0.08749	6.44115	0.01582
20 kGy	30.37944	1.00924 × 10^−4^	81.34161	<0.0001	13.04047	0.0019	14.95515	0.00121

For distilled water (DW), tap water (TW), Sol. A and Sol. B, the results were compared based on the irradiation dose (10 kGy vs. 15 kGy vs. 20 kGy). Similarly, for 10 kGy, 15 kGy and 20 kGy, the results were compared based on the type of water (DW vs. TW vs. Sol. A vs. Sol. B). For DW, TW, Sol. A and Sol. B: number of groups, k = 3 (irradiation doses: 10, 15 and 20 kGy), number of samples, *n* = 9 (3 samples for each irradiation dose), degrees of freedom in the numerator, df1 = 2, and degrees of freedom in the denominator df2 = 6; *F*-critical = 5.1433. For 10 kGy, 15 kGy and 20 kGy: number of groups, k = 4 (DW, TW, Sol. A and Sol. B), number of samples, n = 12 (3 samples for each type of swelling media), degrees of freedom in the numerator, df1 = 3, and degrees of freedom in the denominator df2 = 8; *F*-critical = 4.0662.

**Table 4:** Changes in mass loss from 60 days compared to 30 days, expressed as percentages.

Dose (kGy)	Sol. A (pH = 5.4)	Distilled Water (pH = 6.05)	Sol. B (pH = 7.45)	Tap Water (pH = 7.66)
0% PP (type I)				
10	75.25	24.11	15.77	32.93
15	63.68	52.65	17.76	51.96
20	25.34	93.09	3.75	67.45
0.1% PP (type II)				
10	49.20	28.20	4.90	7.39
15	54.30	34.98	15.89	42.62
20	22.24	94.04	47.24	57.65

**Table 5:** Hydrogel cross-link densities (q) after 30 and 60 days and their modification, expressed as percentages, compared to non-degraded samples.

Dose (kGy)	Sol. A (pH = 5.4)	DW (pH = 6.05)	Sol. B (pH = 7.45)	TW (pH = 7.66)	Sol. A (pH = 5.4)	DW (pH = 6.05)	Sol. B (pH = 7.45)	TW (pH = 7.66)
	q × 10^3^ (mol/cm^3^)				Change (%)			
	30 Days							
	0% PP (Type I)							
10	0.421 ± 0.02 ^bC^	0.710 ± 0.01 ^cA^	0.734 ± 0.01 ^cA^	0.656 ± 0.01 ^cB^	−49.88	−15.48	−12.62	−21.90
15	0.448 ± 0.01 ^bC^	1.085 ± 0.03 ^bA^	0.946 ± 0.03 ^bB^	1.003 ± 0.04 ^bB^	−76.53	−43.16	−50.45	−47.46
20	2.517 ± 0.07 ^aB^	1.926 ± 0.05 ^aC^	2.726 ± 0.07 ^aA^	1.559 ± 0.04 ^aD^	−74.72	−80.66	−72.63	−84.34
	**0.1% PP (Type II)**							
10	0.801 ± 0.01 ^cB^	1.027 ± 0.02 ^cA^	1.012 ± 0.03 ^cA^	0.989 ± 0.02 ^cA^	−28.29	−8.06	−9.40	−11.46
15	1.001 ± 0.04 ^bC^	1.739 ± 0.05 ^bA^	1.521 ± 0.06 ^bB^	1.636 ± 0.04 ^bAB^	−66.16	−41.21	−48.58	−44.69
20	1.777 ± 0.06 ^aC^	2.022 ± 0.05 ^aB^	2.308 ± 0.08 ^aA^	1.972 ± 0.05 ^aB^	−73.75	−70.13	−65.91	−70.87
	**60 days**							
	**0% PP (Type I)**							
10	0.287 ± 0.01 ^cC^	0.683 ± 0.01 ^cA^	0.702 ± 0.02 ^cA^	0.619 ± 0.01 ^cB^	−65.83	−18.69	−16.43	−26.31
15	0.430 ± 0.011 ^bC^	0.871 ± 0.02 ^bB^	0.836 ± 0.02 ^bB^	0.962 ± 0.02 ^bA^	−77.48	−54.37	−56.21	−49.61
20	2.506 ± 0.07 ^aA^	1.306 ± 0.03 ^aC^	2.224 ± 0.06 ^aB^	1.334 ± 0.02 ^aC^	−74.83	−86.88	−77.67	−86.60
	**0.1% PP (Type II)**							
10	0.769 ± 0.02 ^cB^	0.982 ± 0.03 ^cA^	0.990 ± 0.02 ^cA^	0.936 ± 0.02 ^cA^	−31.15	−12.09	−11.37	−16.20
15	0.893 ± 0.02 ^bD^	1.668 ± 0.04 ^bA^	1.400 ± 0.03 ^bC^	1.520 ± 0.04 ^bB^	−69.81	−43.61	−52.67	−48.61
20	1.680 ± 0.05 ^aB^	2.008 ± 0.05 ^aA^	2.037 ± 0.05 ^aA^	1.654 ± 0.04 ^aB^	−75.18	−70.34	−69.91	−75.57

Values within each column for each data set with different lower letters superscripts are significantly different (*p* ≤ 0.05). Values within each row for each data set with different upper letters superscripts are significantly different (*p* ≤ 0.05).

**Table 6:** Hydrogel mesh sizes (q) after 30 and 60 days and their modification, expressed as percentages, compared to non-degraded samples.

Dose (kGy)	Sol. A (pH = 5.4)	DW (pH = 6.05)	Sol. B (pH = 7.45)	TW (pH = 7.66)	Sol. A (pH = 5.4)	DW (pH = 6.05)	Sol. B (pH = 7.45)	TW (pH = 7.66)
	ξ (nm)				Change (%)			
	30 Days							
	0% PP (Type I)							
10	224.27 ± 7.07 ^aA^	155.70 ± 2.08 ^aBC^	152.06 ± 2.03 ^aC^	164.57 ± 2.54 ^aB^	+61.58	+12.18	+9.55	+18.57
15	215.37 ± 4.43 ^aA^	116.12 ± 2.13 ^bC^	127.80 ± 2.51 ^bB^	122.68 ± 3.00 ^bBC^	+175.06	+48.30	+63.22	+56.68
20	66.63 ± 1.29 ^bC^	80.29 ± 1.50 ^cB^	63.01 ± 1.16 ^cD^	93.07 ± 1.48 ^cA^	+160.27	+213.63	+146.13	+263.55
	**0.1% PP (Type II)**							
10	152.46 ± 0.02 ^aA^	128.17 ± 1.43 ^aB^	129.57 ± 2.58 ^aB^	131.60 ± 1.89 ^aB^	+25.69	+5.66	+6.82	+8.49
15	128.32 ± 3.77 ^bA^	87.24 ± 1.58 ^bC^	95.82 ± 2.56 ^bB^	91.06 ± 1.69 ^bBC^	+112.45	+44.44	+58.64	+50.76
20	84.18 ± 2.06 ^cA^	76.89 ± 1.27 ^cB^	70.13 ± 1.62 ^cC^	78.25 ± 1.40 ^cB^	+152.79	+130.90	+110.60	+134.98
	**60 days**							
	**0% PP (Type I)**							
10	293.48 ± 7.17 ^aA^	159.89 ± 2.18 ^aC^	156.84 ± 2.97 ^aC^	171.39 ± 2.65 ^aB^	+111.44	+15.19	+13.00	+23.48
15	221.60 ± 4.26 ^bA^	135.34 ± 2.27 ^bB^	139.30 ± 1.93 ^bB^	126.26 ± 1.81 ^bC^	+183.01	+72.85	+77.91	+61.25
20	66.83 ± 1.38 ^cC^	105.32 ± 1.59 ^cA^	72.63 ± 1.36 ^cB^	103.75 ± 1.35 ^cA^	+161.05	+311.41	+183.71	+305.27
	**0.1% PP (Type II)**							
10	156.97 ± 2.49 ^aA^	132.35 ± 2.68 ^aB^	131.54 ± 2.00 ^aB^	136.78 ± 1.98 ^aB^	+29.41	+9.11	+8.44	+12.76
15	138.98 ± 2.43 ^bA^	89.80 ± 1.32 ^bD^	101.47 ± 1.37 ^bB^	95.83 ± 1.54 ^bC^	+130.10	+48.68	+68.00	+58.66
20	87.53 ± 1.69 ^cB^	77.28 ± 1.42 ^cB^	76.51 ± 1.31 ^cA^	88.48 ± 1.60 ^cA^	+162.85	+132.07	+129.76	+165.71

Values within each column for each data set with different lower letters superscripts are significantly different (*p* ≤ 0.05). Values within each row for each data set with different upper letters superscripts are significantly different (*p* ≤ 0.05).

**Table 7:** Hydrogel swelling at equilibrium (*S_eq_*) after 30 and 60 days and their modification, expressed as percentages, compared to non-degraded samples.

Dose (kGy)	Sol. A (pH = 5.4)	DW (pH = 6.05)	Sol. B (pH = 7.45)	TW (pH = 7.66)	Sol. A (pH = 5.4)	DW (pH = 6.05)	Sol. B (pH = 7.45)	TW (pH = 7.66)
	*S_eq_* (%)				Change (%)			
	30 Days							
	0% PP (Type I)							
10	30,948 ± 835 ^aA^	22,652 ± 259 ^aBC^	22,199 ± 253 ^aC^	23,751 ± 314 ^aB^	+48.70	+8.84	+6.66	+14.12
15	30,576 ± 538 ^aA^	18,028 ± 283 ^bC^	19,569 ± 328 ^bB^	18,895 ± 396 ^bBC^	+126.51	+33.55	+44.97	+39.97
20	14,106 ± 233 ^bC^	16,546 ± 264 ^cB^	13,448 ± 212 ^cD^	18,774 ± 255 ^bA^	+139.41	+180.82	+128.24	+218.64
	**0.1% PP (Type II)**							
10	35,071 ± 535 ^aA^	30,233 ± 289 ^aB^	30,514 ± 519 ^aB^	30,923 ± 380 ^aB^	+52.03	+31.05	+32.27	+34.05
15	26,731 ± 671 ^bA^	19,218 ± 297 ^bC^	20,823 ± 476 ^bB^	19,935 ± 317 ^bBC^	+107.36	+49.08	+61.53	+54.64
20	16,034 ± 336 ^cA^	14,839 ± 210 ^cB^	13,715 ± 271 ^cC^	15,061 ± 230 ^cB^	+121.22	+104.73	+89.22	+107.80
	**60 days**							
	**0% PP (Type I)**							
10	38,953 ± 814 ^aA^	23,173 ± 271 ^aC^	22,794 ± 369 ^aC^	24,591 ± 325 ^aB^	+87.16	+11.34	+9.52	+18.15
15	31,332 ± 515 ^bA^	20,551 ± 295 ^bB^	21,065 ± 249 ^bB^	19,367 ± 237 ^cC^	+132.11	+52.24	+56.05	+43.47
20	14,142 ± 250 ^cC^	20,868 ± 269 ^bA^	15,185 ± 243 ^cB^	20,602 ± 230 ^bA^	+140.02	+254.18	+157.72	+249.66
	**0.1% PP (Type II)**							
10	35,956 ± 487 ^aA^	31,073 ± 538 ^aB^	30,913 ± 403 ^aB^	31,963 ± 396 ^aB^	+55.86	+34.70	+34.00	+38.55
15	28,621 ± 429 ^bA^	19,698 ± 247 ^bD^	21,869 ± 253 ^bB^	20,825 ± 287 ^bC^	+122.02	+52.80	+69.65	+61.55
20	16,578 ± 273 ^cA^	14,902 ± 234 ^cB^	14,776 ± 216 ^cB^	16,732 ± 259 ^cA^	+128.73	+105.60	+103.86	+130.85

Values within each column for each data set with different lower letters superscripts are significantly different (*p* ≤ 0.05). Values within each row for each data set with different upper letters superscripts are significantly different (*p* ≤ 0.05).

**Table 8:** Modifications in cross-link density (q), mesh size (**ξ**) and swelling at equilibrium (*S_eq_*) after 60 days compared to 30 days.

Dose (kGy)	Sol. A (pH = 5.40)			Distilled Water (pH = 6.05)			Sol. B (pH = 7.45)			Tap Water (pH = 7.66)		
	q	ξ	*S_eq_*	q	ξ	*S_eq_*	q	ξ	*S_eq_*	q	ξ	*S_eq_*
	**0% PP (type I)**											
10	−31.83	30.86	25.87	−3.80	2.69	2.30	−4.36	3.14	2.68	−5.64	4.14	3.54
15	−4.02	2.89	2.47	−19.72	16.55	13.99	−11.63	9.00	7.64	−4.09	2.92	2.50
20	−0.44	0.30	0.26	−32.19	31.17	26.12	−18.42	15.27	12.92	−14.43	11.48	9.74
	**0.1% PP (type II)**											
10	−4.00	2.96	2.52	−4.38	3.26	2.78	−2.17	1.52	1.31	−5.36	4.17	3.36
15	−10.79	8.31	7.07	−4.08	2.93	2.50	−7.96	5.90	5.02	−7.09	5.24	4.46
20	−5.46	3.98	3.39	−0.69	0.51	0.42	−11.74	9.10	7.74	−16.13	13.07	11.09

**Table 9:** The positions of the ν(CH) bonds and the Δν values in solutions of different pH at 30 and 60 days of degradation.

Hydrogel Type	Frequency of ν(CH) Band (cm^−1^) at Different pH					Δν (cm^−1^)			
	Native	Sol. A	DW	Sol. B	TW	Sol. A	DW	Sol. B	TW
Type I (0% PP)	2932	2937 ^a^	2941 ^a^	2933 ^a^	2935 ^a^	5 ^a^	9 ^a^	1 ^a^	3 ^a^
		2939 ^b^	2937 ^b^	2937 ^b^	2941 ^b^	7 ^b^	5 ^b^	5 ^b^	9 ^b^
Type II (0.1% PP)	2925	2940 ^a^	2936 ^a^	2938 ^a^	2938 ^a^	15 ^a^	11 ^a^	13 ^a^	13 ^a^
		2938 ^b^	2936 ^b^	2938 ^b^	2939 ^b^	13 ^b^	11 ^b^	13 ^b^	14 ^b^

^a^—degradation for 30 days in pH solution. ^b^—degradation for 60 days in pH solution.

**Table 10:** Physico-chemical properties of hydrogels used in the swollen state for the burial in soil experiments: q, cross-link density (mol/cm^3^); ξ, mesh size (nm); and *S_eq_*, swelling at equilibrium.

Dose (kGy)	Cross-Link Density q × 10^3^ (mol/cm^3^)	Mesh Size ξ (nm)	Swelling at Equilibrium *S_eq_* (%)
**0% PP (Type I)**			
10	0.840 ± 0.07	138.8 ± 7.96	20,813 ± 1154
15	1.909 ± 0.09	78.3 ± 5.54	13,499 ± 388
20	9.958 ± 0.82	25.6 ± 1.44	5892 ± 374
**0.1% PP (Type II)**			
10	1.117 ± 0.11	121.3 ± 8.25	23,069 ± 1060
15	2.958 ± 0.24	60.4 ± 3.24	12,891 ± 802
20	6.770 ± 0.86	33.3 ± 2.86	7248 ± 533

**Table 11:** Relevant specifications of distilled water, tap water, Sol. A and Sol. B.

Swelling Media	Relevant Specifications
Distilled water	pH: 6.05
Tap water	NH^4+^ < 0.05 mg/L; Ca^2+^ = 58.44 mg/L; Mg^2+^ = 35.44 mg/L; NO^2−^ < 0.033 mg/L; NO^3−^ = 5.02 mg/L; pH = 7.66
Synthetic nutrient solution (Sol. A)	Liquid fertilizer for balcony flowers, produced by AGRO CS, Lucenec, Slovakia, was used according to the manufacturer’s instructions, 7.5 mL diluted in 1000 mL of distilled water. Total nitrogen: 3.6%; NO_3_^−^ = 1.8%; NH_4_^+^ = 1.8%; P_2_O_5_ = 2.3%; K_2_O = 2.7%; pH = 5.40 [37,38,49].
100% natural organic nutrient solution (Sol. B)	Biopon, natural biohumus for vegetables and greens flowers, produced by Bros Sp. z o.o. sp. k., Poznan, Poland, was used according to the manufacturer’s instructions, 60 mL diluted in 1000 mL of distilled water. Total nitrogen: 0.02%; P_2_O_5_ = 0.02%; K_2_O = 0.05%; organic matter = 40.0%; pH = 7.45 [37,38,49].

**Table 12:** Relevant specifications of the soil used in the degradation experiments.

Soil Type	Agro-Technical Features
Universal soil for flowers, houseplants, planters, greenhouses and seedlings with high organic matter content (Florimax, produced by S.C. Biofit Exim S.R.L., Bucharest, Romania)	organic matter = 29–60%, mineral salts = 0.35–0–75%, mineral nitrogen = 25–50 ppm, potassium (water-soluble) = 52–85 ppm, phosphorus (soluble) = 32–55 ppm, pH neutral

## Data Availability

All experimental data are presented in the article.
